# Supplementary material for: High-performance silk-based hybrid membranes employed for osmotic energy conversion
Source: Nat Commun. 2019 Aug 28;10:3876. doi: 10.1038/s41467-019-11792-8 (PMC6713777; doi:10.1038/s41467-019-11792-8)
Supplement: Supplementary file 1 — Supplementary Information [file 41467_2019_11792_MOESM1_ESM.pdf]

Supplementary Information

High-performance silk-based hybrid membranes employed for osmotic energy conversion

Xin *et al.*

Supplementary Figures

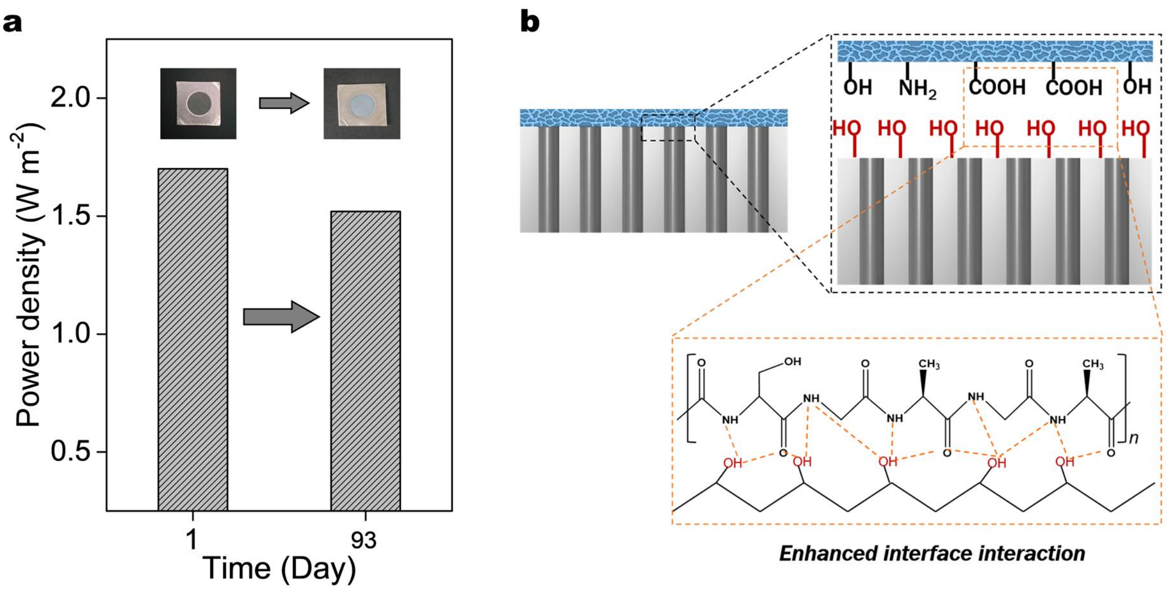

Supplementary Figure 1. The stability of the AAO/SNF membrane. **a** The power densities of the hybrid membrane after immerging different days. Insets: optical images of the hybrid membrane. The membrane shows no separation. **b** The schematically illustrating the reinforcement of interface between SNF and AAO membranes.

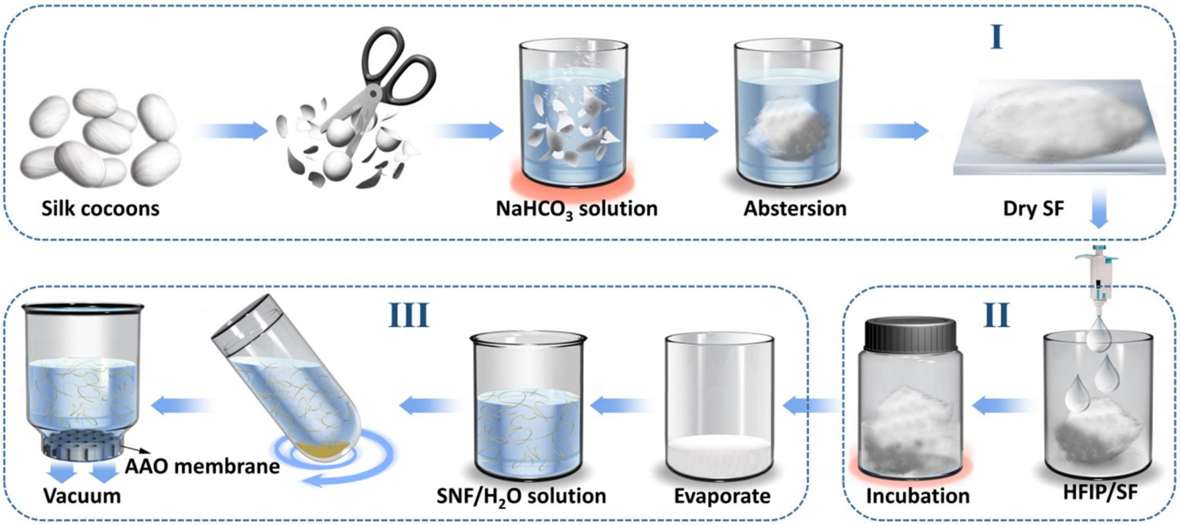

Supplementary Figure 2. The detailed treatment route for fabricating the hybrid membrane. In step I, 0.5 wt% sodium bicarbonate ($NaHCO_3$) solution is firstly prepared by dissolving 10.00 g of $NaHCO_3$ in 2-liters of deionized water and heated until boiling. *Bombyx mori* cocoon is cut into finger-shaped pieces and degummed twice in the boiled $NaHCO_3$ solution for 30 min. Then the degummed silk fibers are thoroughly washed for three times with the cold deionized water. Then, the silks are spread on a new piece of aluminum foil and dried in air at room temperature overnight. In step II, the degummed silk is well mixed with HFIP with a weight ratio of 1:30 (silk fibers/HFIP), and the mixture is incubated at 60°C for 24 h with airtight container. In step III, the silk microfibrils pulp is dried in a fuming cupboard to evaporate HFIP for approximately 5 h, followed by adding deionized water with a weight ratio of 1:400 under continuous stirring. The undissolved materials are removed manually. The microfibrils/water mixture is sonicated at 40 kHz frequency for 60 min, the exfoliated SNF dispersion (0.05 wt%) is collected by centrifugation at 10000 rpm for 30 min. Finally, the hybrid membrane could be obtained through vacuum filtration of the SNF solution on AAO substrate.

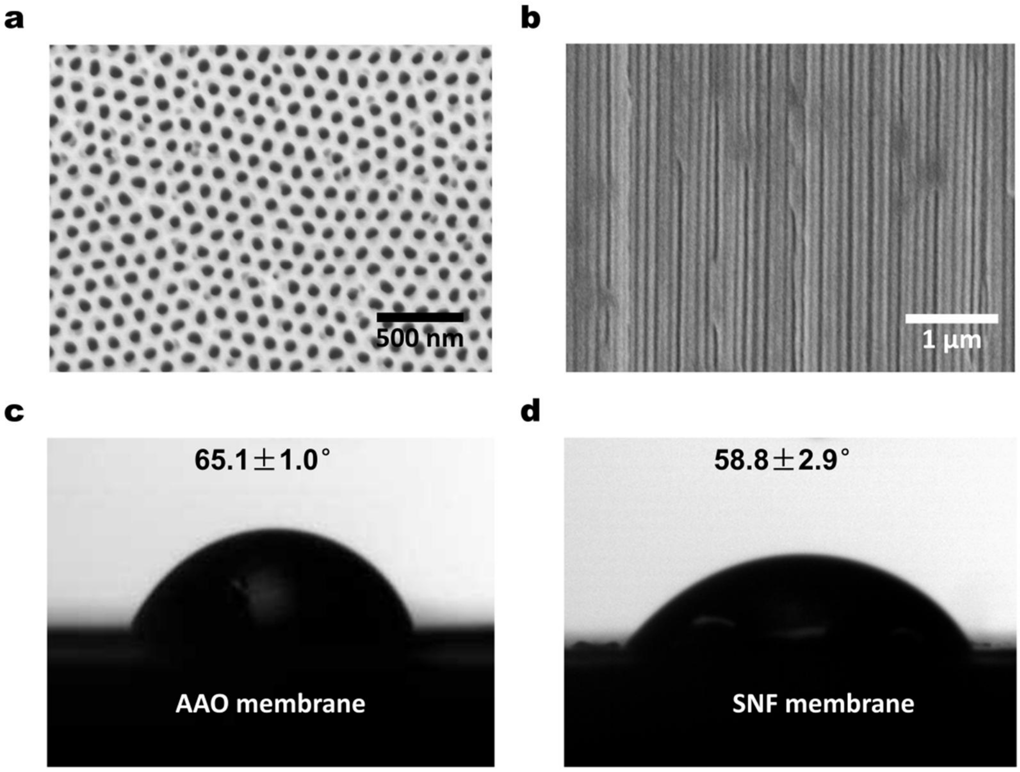

Supplementary Figure 3. Characterization of the hybrid membrane. SEM images of the AAO membrane. The top view (**a**) and the cross-sectional (**b**) view of the AAO shows the channel size of ~80 nm, respectively. Contact angle measurement of the AAO (**c**) and the SNF membranes (**d**), respectively. Error bars represent s.d.

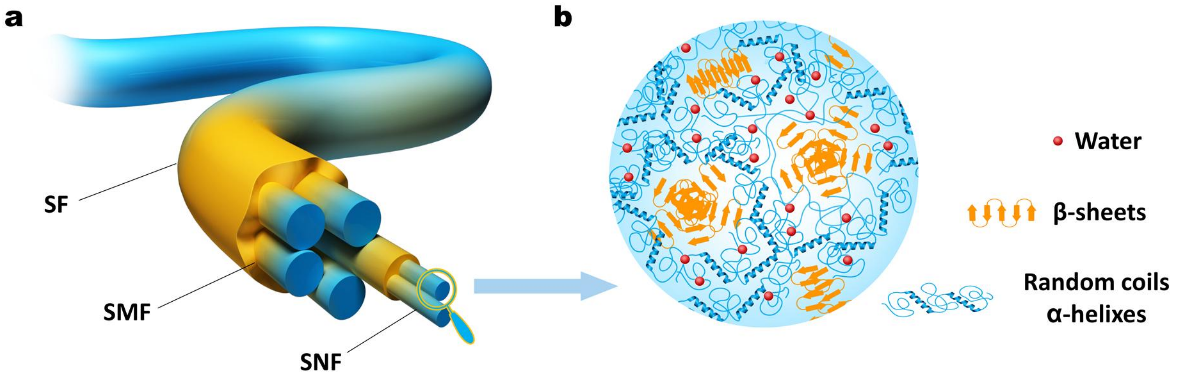

Supplementary Figure 4. The hierarchical structure of *Bombyx mori* silkworm. **a** Silk fibers (SF) are composed of several silk microfibers (SMF) including numerous silk nanofibrils (SNF). **b** Enlarged schematic of one single SNF. The protein is composed of stacked β-sheets (yellow arrows), helical α-helixes (blue helix), and random coils (blue coils). Water molecules (red ball) mainly interact with random coils.

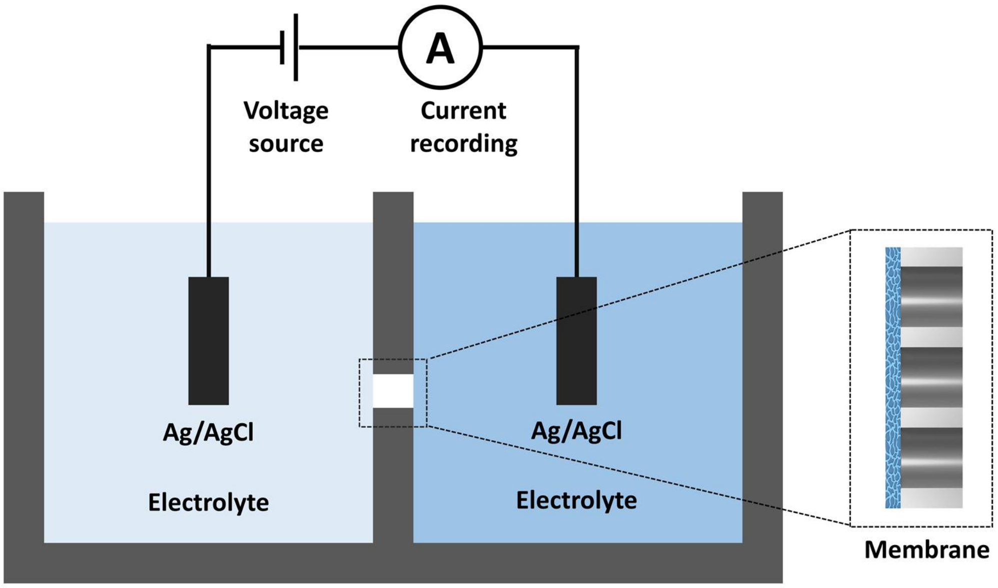

Supplementary Figure 5. Schematic of the home-made conductance cell. The ionic transport property of the hybrid membrane is monitored by recording the ionic current through the hybrid membrane. The ionic current is measured by a Keithley 6487 picoammeter (Keithley Instruments, Cleveland, OH). The hybrid membrane is mounted in between a two-compartment electrochemical cell.

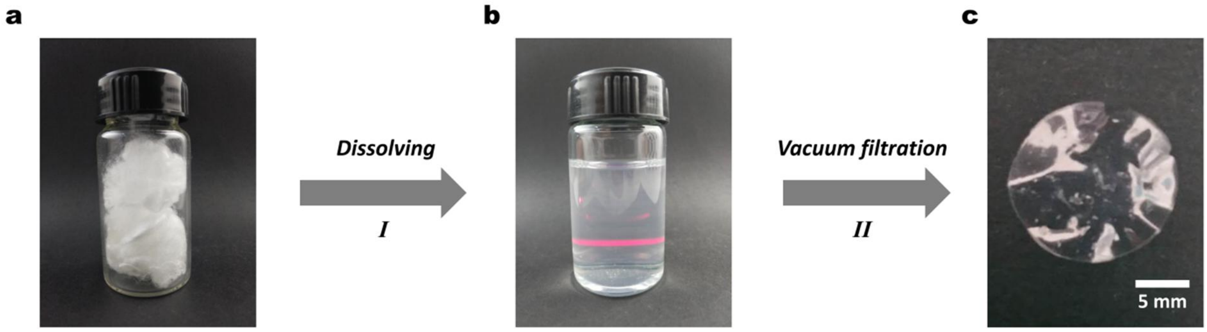

Supplementary Figure 6. The preparation process of the SNF membranes. The degummed *B. mori* silk fibers (**a**) are dissolved to show a uniform solution (**b,** Tyndall scattering). With the aid of vacuum filtration, the free-standing SNF membrane (**c**) is formed.

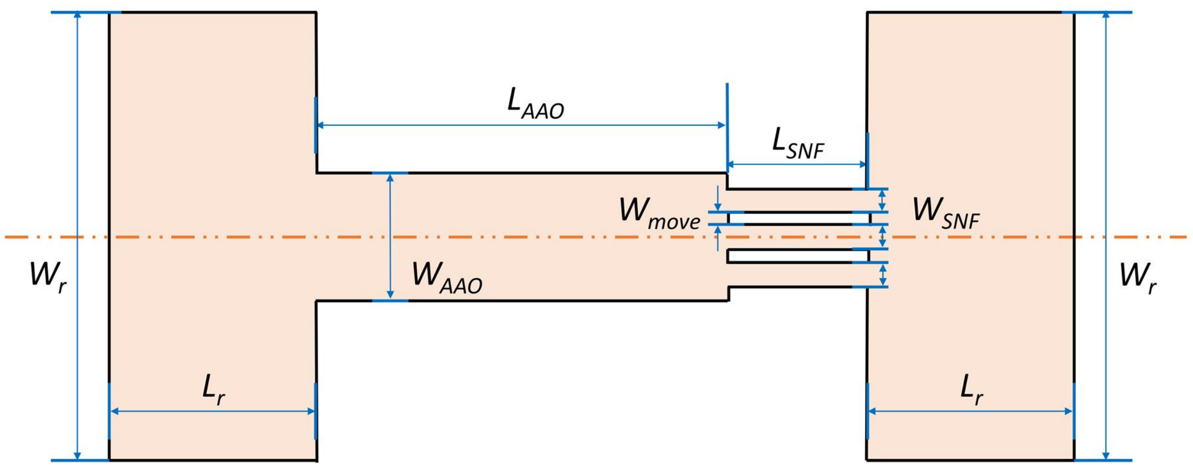

Supplementary Figure 7. Schematic of the numerical simulated model. The 3D models (shown as a cutplane) contain a combined channel connected by two electrolyte reservoirs with one 60-µm AAO channel and three 10-µm SNF membrane channels. Two electrolyte reservoirs are introduced to reduce the effect of entrance/exitn mass transfer resistances (Drawing not to scale).

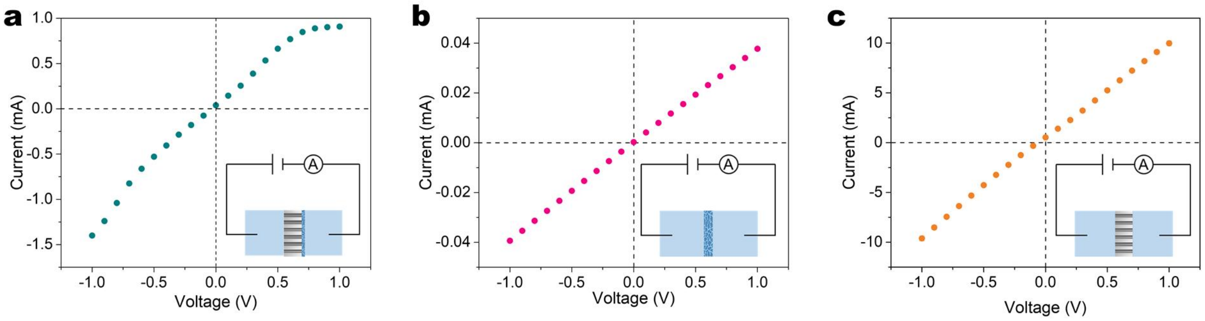

Supplementary Figure 8. The *I-V* curves of three types of membranes (AAO/SNF hybrid membrane, SNF membrane, and AAO membrane). **a** The *I-V* curve of the hybrid membrane in 0.1 M KCl solution shows rectification. **b** The *I-V* curve of SNF membrane in 0.01 M KCl solution. **c** The *I-V* curve of AAO membrane in 0.01 M KCl solution.

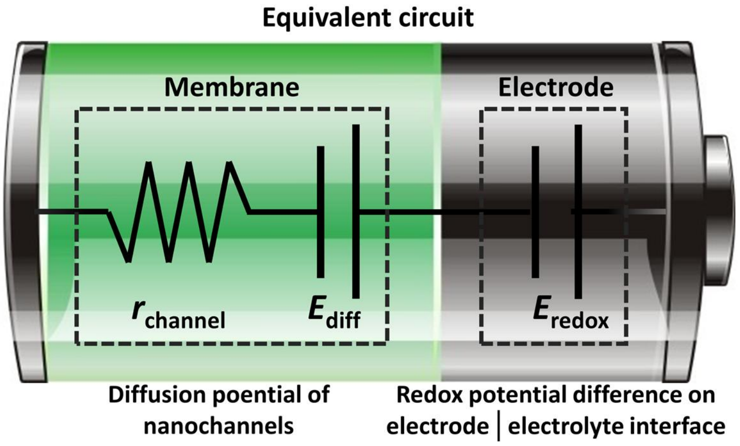

Supplementary Figure 9. Equivalent circuit diagram of the power source. The measured V_{OC} is composed of two parts, E_{redox} and E_{diff}.

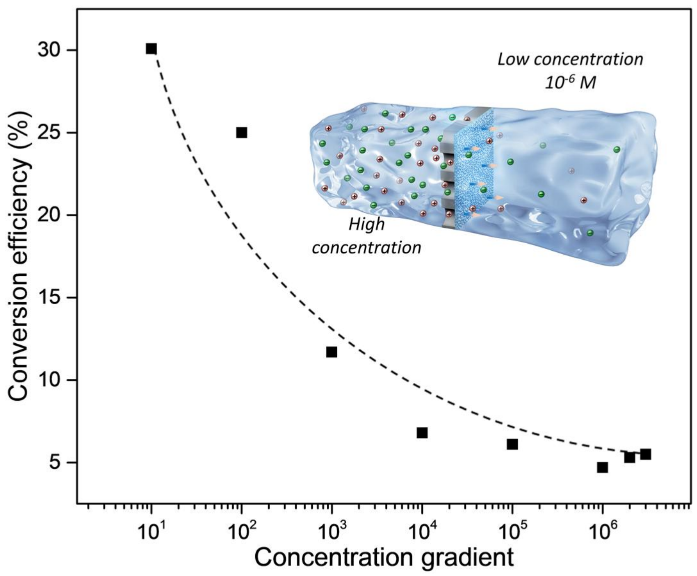

Supplementary Figure 10. The relationship between the energy conversion efficiency and concentration gradient. The KCl concentration on the SNF membrane side is fixed to 1 μM; the KCl concentration on the AAO membrane side, which gradually increases from 1 μM to 3 M. The corresponding energy conversion efficiency declines from 30.1% to 5.5% (The dash line is for the guide of eye sight).

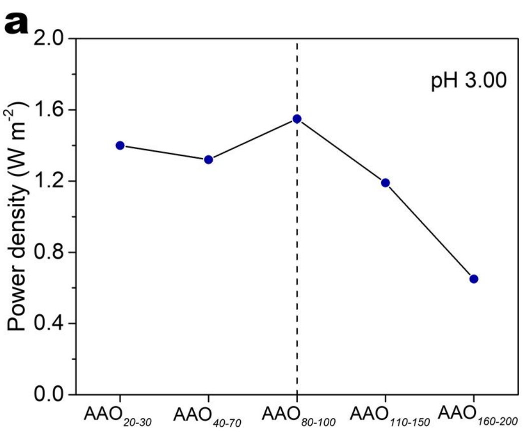

Supplementary Figure 11. Effect of AAO channel size on the power density at pH 3.00 and pH 11.00. Both the power densities at pH 3.00 (**a**) and pH 11.00 (**b**) reach the maximum values with the 80-100 nm AAO channels size. Also, the hybrid membrane shows better energy conversion in basic condition (pH 11.00) with the power density up to 2.86 W m^{-2}.

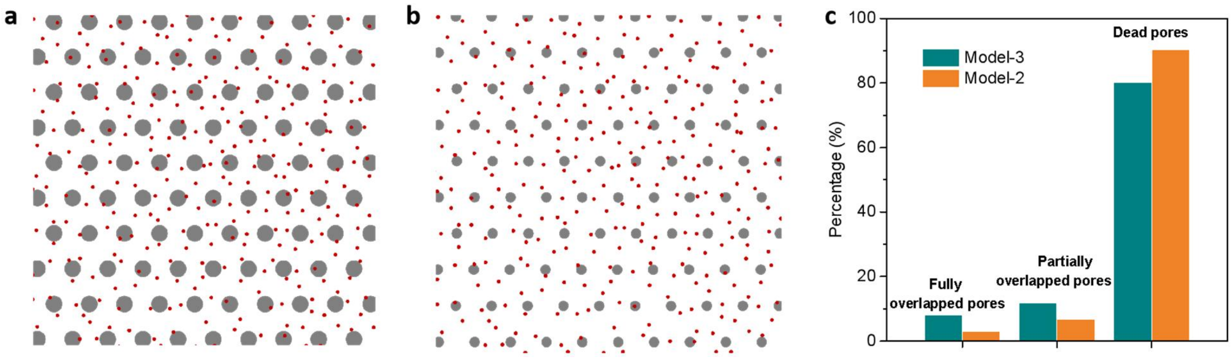

Supplementary Figure 12. Statistics analysis of the amount of the fully overlapped pores, partially overlapped pores, and dead pores. We first develop two arrays of pores mimicking the two membrane layers. The pores of the two layers are assumed to be perfect rounded packed distribution. The geometry parameters are set according to the AFM images. For the AAO membrane, the pore size is set to 90 nm and the center-to-center distance is set to 190 nm. For the SNF membrane, the pore size is set to 20 nm and randomly generated an unselected pore distribution. The porosity of AAO membrane is consistent, and the random distribution of SNF pores is the same. The ratio of these three types of pores for Model (3) (which is consistent with Fig. 4 (3), and the AAO channels are 80-100 nm.) is approximately 8.0%, 11.7%, 80.3%, respectively (**a**, **c**). The ratio of these three types of pores for Model (2) (which is consistent with Fig. 4 (2), and the AAO channels are 40-70 nm.) is approximately 3.0%, 6.7%, 90.3%, respectively (**b**, **c**), both whose fully and partially overlapped pores are further fewer than that of Model (3).

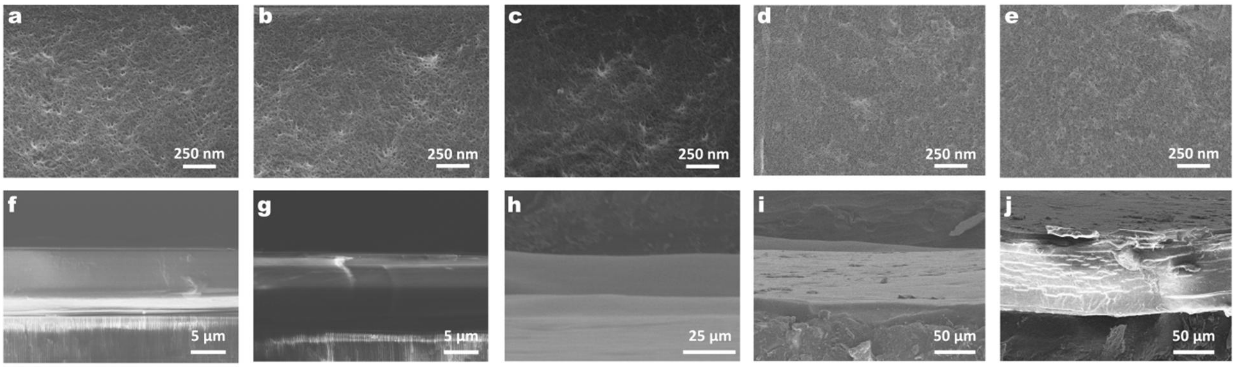

Supplementary Figure 13. SEM images of SNF membranes. (**a**, **f**) SNF membranes prepared by 5 mL SNF dispersion with a concentration of 0.05 wt%. (**b**, **g**) SNF membranes prepared by 10 mL SNF dispersion with a concentration of 0.05 wt%. (**c**, **h**) SNF membranes prepared by 15 mL SNF dispersion with a concentration of 0.05 wt%. (**d**, **i**) SNF membranes prepared by 20 mL SNF dispersion with a concentration of 0.05 wt%. (**e**, **j**) SNF membranes prepared by 25 mL SNF dispersion with a concentration of 0.05 wt%. (**a**-**e**) surface image of the membranes. (**f**-**j**) cross-section images of the membranes. The thicknesses of (**f**-**j**) are 5 µm, 10 µm, 15 µm, 40 µm, and 80 µm, respectively.

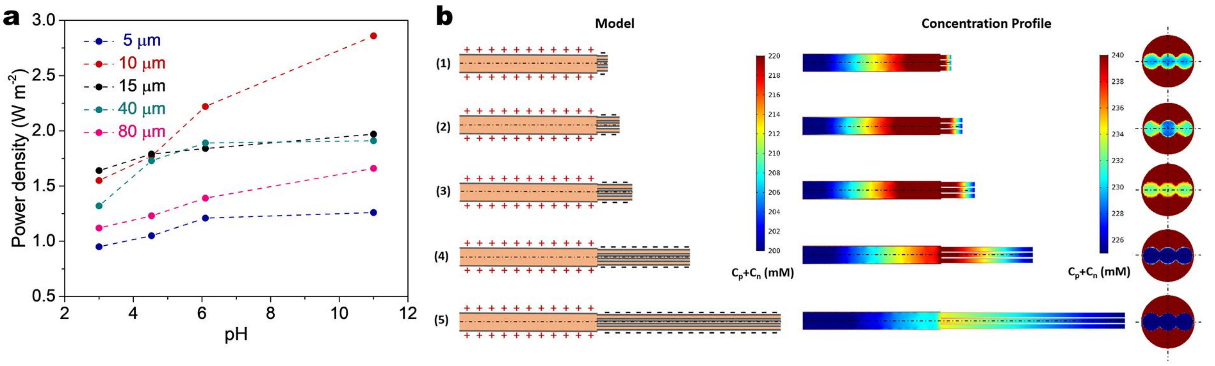

Supplementary Figure 14. Effect of the SNF membrane thickness on the energy conversion. **a** Experimental power densities of the hybrid membranes with different thickness of SNF membranes (5 μm, 10 μm, 15 μm, 40 μm, and 80 μm) at different pH values. **b** Corresponding simulated ion concentration profiles based on 3D models of hybrid membranes with different SNF membranes thickness (5 μm, 10 μm, 15 μm, 40 μm, and 80 μm). In all simulation, the applied bias is set to 1 V. The different sectional concentration profiles clearly show that the hybrid membranes with 10- and 15-μm-thickness SNF membrane hold larger ion accumulation.

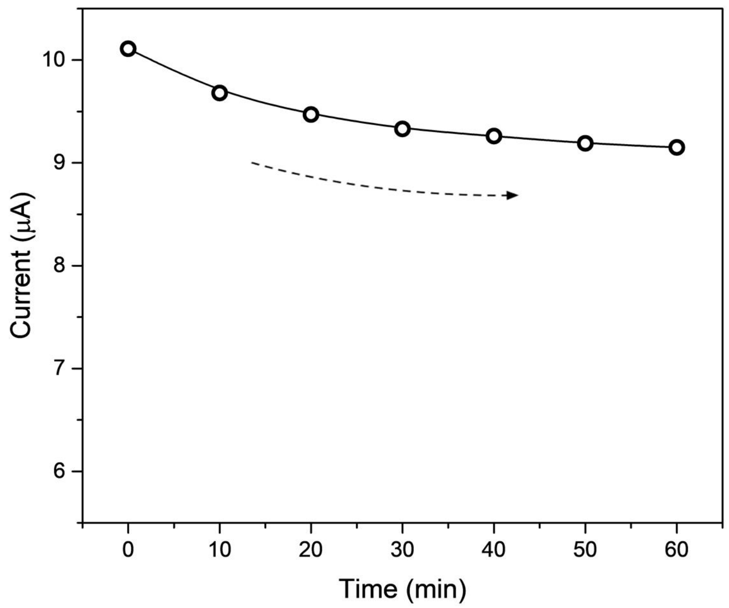

Supplementary Figure 15. Current-time (*I-T*) curve of the hybrid membrane. The concentration gradient of 0.1 M/1 μM is used in the measurement. The diffusion current across the membrane slowly attenuates, primarily due to the dissipated concentration gradient, and sort of exposure to air and absorption of CO_2.

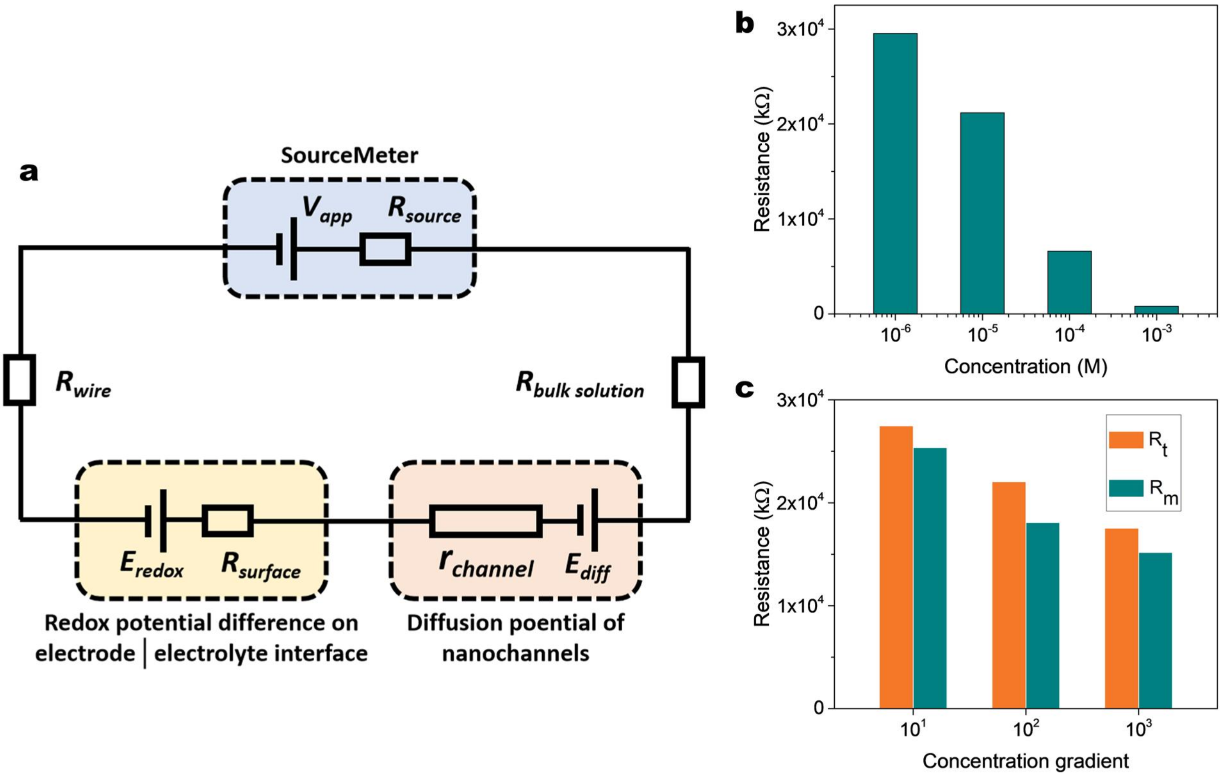

Supplementary Figure 16. The contribution of various resistances in the system. **a** The equivalent circuit diagram of the power source, where E_{diff} and $r_{channel}$ represent the open-circuit voltage and the corresponding inner resistance of channels, respectively. R_t is the total resistance of the system, including the resistance of the membrane ($r_{channel}$), the inner resistance (R_{source}) of voltage source (V_{app}), the resistance ($R_{surface}$) existing in the interfaces of electrodes, the tandem resistance (R_{wire}) of the wires and the electrolyte, and the bulk solution resistance ($R_{bulk\ solution}$); also R_m, the total resistance of multiple components in the circuit excluding the membrane resistance. R_m can be calculated by the equation:

$$R_m = \frac{1}{2}(R_H + R_L)$$

where R_H and R_L refer to the corresponding resistances in high-concentration and low-concentration solution, respectively, which have been measured (**b**). Then, the R_m in different concentration gradients is calculated and compared with R_t. (**c**) Along with the concentration gradient increasing, both the R_t and the R_m gradually decreases, and the R_t is mainly contributed by R_m. Thus, by bringing down the R_m, the output power density could be further increased.

Supplementary Tables

Supplementary Table 1. The corresponding values of V_{OC}, E_{redox}, and E_{diff} under different concentration gradient conditions.

Concentration gradient (M/M)	$10^{-6}/10^{-5}$	$10^{-6}/10^{-4}$	$10^{-6}/10^{-3}$	$10^{-6}/10^{-2}$	$10^{-6}/10^{-1}$	$10^{-6}/1$	$10^{-6}/2$	$10^{-6}/3$	0.01/0.5
V_{OC} (mV)	49	110	139	148	212	236	250	263	93
E_{redox} (mV)	4	28	55	62	110	129	131	138	35
E_{diff} (mV)	45	82	84	86	102	107	119	125	58

Supplementary Table 2. Possible sources of saline solutions for energy conversion by RED (partial information obtained from https://doi.org/10.1016/B978-0-08-100312-1.00005-5). As shown in the table, the pH of these resources is mainly distributed in the alkaline range. Specially, our system shows great potentials for using highly saline and alkaline waste solutions from industrial activities (highlighted in yellow).

Sources	Typical concentration	Volumes availability	Geographical constrains	pH
**Brines**				
Saltworks (seasalt production facilities)	>200 g/L up to saturation	Low/medium	Site specific	7-9
Natural salt ponds	>200 g/L up to saturation	Low/medium	Site specific	~9
Salt lakes	>250 g/L up to saturation	Medium/large	Very site specific	~9
Industrial activities	From 50 to 300 g/L	Low/medium	Industrial sites	4-13
Mining activities	From 100 to 300 g/L	Low/medium	Site specific	7-9
Desalination brines	From 50 to 100 g/L	Medium	Site specific	7.5-8.5
**Saline waters**				
Seawater	From 20 to 50 g/L	Very large	Wide-spread (in coastal areas)	8-8.5
Brackish water	From 2 to 20 g/L	Medium/large	Quite wide-spread	~8
Textile industry	From 1 to 20 g/L	Low	Industrial sites	5-14
Waste water from oil refining activities	From 5 to 10 g/L	Low	Refinery sites	7.5-8
**Freshwater**				
Lakes	<1 g/L	Large	Slightly site specific	7.8-8.5
Rivers	<1 g/L	Large	Slightly site specific	6-8
Effluents from wastewater treatment plant	<1 g/L	Medium	Slightly site specific	6-9

Supplementary References

(1) Cao, L. et al. Towards understanding the nanofluidic reverse electrodialysis system: well matched charge selectivity and ionic composition. *Energ. Environ. Sci*. **4,** 2259-2266 (2011).

(2) White, H. S., & Bund, A. Ion current rectification at nanopores in glass membranes. *Langmuir* **24,** 2212-2218 (2008).

(3) Cervera, J., Schiedt, B., Neumann, R., Mafe, S. & Ramirez, P. Ionic conduction, rectification, and selectivity in single conical nanopores. *J. Chem. Phys*. **124,** 104706 (2006).

(4) Kim, D., Duan, C., Chen, Y. & Majumdar, A. Power generation from concentration gradient by reverse electrodialysis in ion-selective nanochannels. *Microfluid. Nanofluid*. **9,** 1215-1224 (2010).

(5) Guo, W. et al. Energy harvesting with single-ion-selective nanopores: a concentration-gradient-driven nanofluidic power source. *Adv. Funct. Mater*. **20,** 1339–1344 (2010).

(6) Rockwood, D. N. et al. Materials fabrication from Bombyx mori silk fibroin. *Nat. Protoc*. **6,** 1612-1620 (2011).

(7) Hong, M.-S. et al. Biomimetic chitin-silk hybrids: an optically transparent structural platform for wearable devices and advanced electronics. *Adv. Funct. Mater*. **28,** 1705480 (2018).

(8) Lin, N. & Liu, X. Y. Correlation between hierarchical structure of crystal networks and macroscopic performance of mesoscopic soft materials and engineering principles. *Chem. Soc. Rev*. **44,** 7881-7915 (2015).

(9) Ling, S., Jin, K., Kaplan, D. L. & Buehler, M. J. Ultrathin free-standing bombyx mori silk nanofibril membranes. *Nano Lett*. **16,** 3795-3796 (2016).

(10) Zhou, Z. et al. Engineering the future of silk materials through advanced manufacturing. *Adv. Mater*. **30,** e1706983 (2018).

(11) Gao, J. et al. High-performance ionic diode membrane for salinity gradient power generation. *J. Am. Chem. Soc*. **136,** 12265-12272 (2014).

(12) Sui, X. et al. Biomimetic nanofluidic diode composed of dual amphoteric channels maintains rectification direction over a wide ph range. *Angew. Chem., Int. Ed*. **55,** 13056-13060 (2016).

(13) Huang, X., Kong, X.-Y., Wen, L. & Jiang, L. Bioinspired ionic diodes: from unipolar to bipolar. *Adv. Funct. Mater*. **28,** 1801079 (2018).

(14) Ouyang, W. et al. Nanofluidic crystal: a facile, high-efficiency and high-power-density scaling up scheme for energy harvesting based on nanofluidic reverse electrodialysis. *Nanotechnology* **24,** 345401 (2013).
